# KG-bench: benchmarking graph neural network algorithms for drug repurposing

**DOI:** 10.1093/bioinformatics/btag159

**Published:** 2026-05-08

**Authors:** Siqi Wei, Christo Sasi, Jelle Piepenbrock, Martijn A Huynen, Peter A C ’t Hoen, SIMPATHIC Consortium, Tessa Braam, Esther Bührman, Juliette Chevalier, Martina Cornel, Lotte Haverman, Vivi Heine, Annelieke Müller, Tessel Rigter, Ignacio Escuder Bueno, Leda Persidi, Eftychia Lekka, Andreas Persidis, Vassilis Virvilis, Markus Schülke-Gerstenfeld, Emily Freeman, Haley Geertsma, Hanns Lochmuller, Alex Mackenzie, Kaela O’Connor, Izabella Pena, Sally Spendiff, Rachel, Martin de Kort, Anna Sanchez, Cansu Tekin, Francisca Vargas Lopes, Larisa Aragon Castro, Mandy Daly, Maria Dutarte, Jana Popova, Enrico Tricanico, Julie Greenfield, Andreas Nadke, Peter Ashley, Alain Geille, Christiana Vasileiadi, Jõao Cardoso, Eduardo Quemada, Mark Wilkinson, Albert Carbonell, Laura Benkemoun, Magda Granata, Pascale Milani, Chloe Morel, Daniel Scherman, Judith Hatzfeld, Selene Lickfett, Alessandro Prigione, Isabella Tolle, Elena Boyd, Samuele Cesaro, Florian Gleich, Samuel Hofmann, Sabine Jung-Klawitter, Stefan Kölker, Christian Thiel, Jo de Bry, Fotis Aisopos, Anastasia Krithara, Anastasios Nentidis, George Paliouras, Stavroula Svolou, Merel Adjobo-Hermans, Hans van Bokhoven, Hilde Braakman, Baziel van Engelen, Alex Garanto, Elena Garcia Lara, Joanna in ’t Hout, Martijn Huijnen, Mirian Janssen, Werner Koopman, Dirk Lefeber, Kris Leeuwenberg, Karlien Mul, Nael Nadif Kasri, Kit Roes, Raymond Schipper, Vedrana Stefanic, Steven Teerenstra, Peter-Bram ’t Hoen, Nicol Voermans, Bart van de Warrenburg, Rick Wansink, Siqi Wei, Ka Man Wu, Jurriaan Zwier, Claudio Cinquemani, Philip Yeske, Adelaide Maria Fernandes Borralho, Maria Martins, Sara Pintado, Rok Dreu, Iztok Grabnar, Jakob Kolar, Zoran Lavric, Igor Locatelli, Irena Mlinaric, brahim Boussaad, Vyron Gorgogietas, Rejko Krueger, Alexia Tiberi, Holm Graessner, Charlotte Kogel, Olaf Riess, Birte Zurek, Priscila Pereira Sena, Thorsten Schmidt, Hanka Dekker

**Affiliations:** Department Medical BioSciences, Radboud University Medical Center, Nijmegen 6525 GA, The Netherlands; Department Medical BioSciences, Radboud University Medical Center, Nijmegen 6525 GA, The Netherlands; Institute for Computing and Information Sciences, Radboud University, Nijmegen 6525 XZ, The Netherlands; Institute for Computing and Information Sciences, Radboud University, Nijmegen 6525 XZ, The Netherlands; Department Medical BioSciences, Radboud University Medical Center, Nijmegen 6525 GA, The Netherlands; Department Medical BioSciences, Radboud University Medical Center, Nijmegen 6525 GA, The Netherlands

## Abstract

**Motivation:**

Drug repurposing leverages existing drugs for new indications, accelerating drug development. Computational methods integrating diverse biological and chemical data can systematically prioritize repurposing candidates, but standardized benchmarks for deep learning evaluation are lacking. We present knowledge graph (KG)-Bench, a graph neural network (GNN) benchmarking framework designed to systematically compare the performance of different GNN architectures on drug-disease association prediction using the Open Targets dataset. We constructed a KG of drugs, diseases, and targets, including annotations such as therapeutic area and molecular pathway, and ensured retrospective validation by leveraging regular dataset updates. To avoid data leakage, we removed redundant entities across splits.

**Results:**

Benchmarking six GNN architectures, Relational Graph Convolutional Networks achieved the highest ranking performance (AUC: 0.91), while TransformerConv showed superior robustness under class imbalance (F1: 0.28 at 1:100 positive: negative ratio), characteristic of real drug repurposing datasets. KG-Bench also assesses bias, node/feature importance, and uses GNNExplainer for interpretability. Our open-source framework enables fair, reproducible evaluation of graph-based drug repurposing algorithms.

**Availability and implementation:**

Data and codes are available at https://github.com/cmbi/Benchmark_GNN_OpenTargets.

## 1 Introduction

Drug repurposing aims to identify new therapeutic uses for existing drugs by leveraging knowledge from previously known drug–target interactions (Ashburn and Thor 2004). Historically, successful drug repurposing projects have been conducted by biologists and pharmaceutical domain experts based on experimental evidence published in literature and clinical trial results (Hoffman-Andrews 2017, Canaud et al. 2023, De Rosa et al. 2023). For rare and previously untreated diseases, insights from domain experts may nevertheless not be enough (Nosengo 2016, Hauser et al. 2017).

Given the interconnected nature of drugs, diseases, targets, and biological pathways, deep learning provides a natural framework for drug repurposing by modeling complex biological relationships and predicting missing links that represent potential therapeutic opportunities. Deep learning methods allow automatic feature learning from massive datasets (Aliper et al. 2016). Furthermore, drug repurposing draws upon diverse data like chemical structures, genomic profiles, and protein–protein interactions. Given the diversity in the innate properties of the entities in biomedical datasets, deep learning frameworks learn from implicit complex relationships despite the variability in data source and data type (Ching et al. 2018). The nonlinear modeling capabilities of deep learning architectures often result in superior predictive performance compared to traditional machine learning methods that rely on handcrafted features or linear models for identifying potential drug repurposing candidates (Zeng et al. 2019).

Graphs can be used for the representation of functional, structural, and other complex information inherent in non-standard real-world data to train a graph neural network (GNN) (Hammer and Jain 2004, Zaripova et al. 2023). A more enriched variant of a graph, which can be used for the representation of knowledge, is called a KG. The cumulative biological and pharmacological knowledge from clinical and molecular interaction studies can be extracted from databases such as ChEMBL (https://www.ebi.ac.uk/chembl/), DrugBank (https://go.drugbank.com/), PubChem (https://pubchem.ncbi.nlm.nih.gov/), and PDB (https://www.rcsb.org/). KGs are composed of nodes, edges, and their features. Nodes are entities labelled with unique keys such as a ChEMBL ID for a molecule, an Experimental Factor Ontology (EFO) ID for a disease, and an Ensembl Gene ID for a gene. Edges are entities used to represent the relationship between two node entities. For example, if a molecule *m* is approved for the treatment of a disease *d*, it can be represented as (m,d). In addition, the properties or characteristics of the nodes and edges (features) are encoded as tensors that can capture and represent the various features. Feature types may include Boolean features, numerical features, categorical features, and text features. By encoding features in different ways, graphs can effectively represent complex entities in a format that models can process. This enables models to learn and distinguish the properties of different nodes, thereby implementing tasks such as classification, prediction, and reasoning in the graph structure (Scarselli et al. 2009). Drug repurposing candidate selection based on biomedical KGs has gained popularity in recent years because they facilitate the representation of intricate biological relationships, such as drug–target interactions, disease–gene associations, shared molecular pathways, and phenotypic similarities between diseases (Wang et al. 2014, Himmelstein et al. 2017).

Graph learning algorithms can be trained on KGs to predict new links between previously unconnected nodes. These methods leverage the topology and features of a graph and iteratively propagate information from a node to its neighbors (Sun et al. 2020). While the complexity of deep learning can hinder interpretation, GNNs offer reasoning opportunities. This contributes to the understanding of mechanisms of action and builds greater confidence in predictions (Stokes et al. 2020). GNNs are expected to make better predictions when the edge connectivity in a graph is dense, as it provides more information for the model to learn from. This is especially true in biomedical KGs where multiple relationships between nodes can offer valuable insights. For instance, when predicting drug–disease associations, GNNs can leverage not only direct drug–target interactions but also indirect pathways through shared protein targets, gene co-expression patterns, and disease phenotype similarities (Doshi and Chepuri 2022).

In a KG-based prediction of drug repurposing candidates, representations of drugs and diseases that are already linked are expected to aid the GNN in predicting additional links for these drugs (Schlichtkrull et al. 2017). The loss function penalizes the model for incorrectly predicting links that do not exist and for missing links that do exist in a validation or test set. A positive link prediction occurs when the model accurately predicts a link that exists in the validation dataset. Conversely, a negative link prediction is when the model correctly predicts the absence of a link that does not exist in the validation dataset. However, biomedical KGs typically contain only observed positive associations (known drug–disease relationships), while the vast majority of possible drug–disease pairs remain unobserved and unlabeled, and are typically sampled from the non-existent links. This creates an imbalanced learning scenario and affects the accuracy and generalization capacity of drug repurposing tasks (Schuler et al. 2022, Hung Le et al. 2024).

The Open Targets platform provides a single source of more than ∼20 publicly available expert-annotated biomedical datasets. The dataset contains some salient features that can be useful in creating a graph representation of the data and a deep learning framework for drug repurposing. The Open Targets dataset is updated every three months with new annotations based on real-world discoveries of gene-disease associations and drug-disease approvals based on successful clinical trials reported in the literature. This allows users, in principle, to train models on historical data and validate and test the models using links added in subsequent updates. Such a train-validate-test framework is useful for drug repurposing predictions. An analysis of the Open Targets Dataset (Ochoa et al. 2022) showed that 33 out of 50 (66%) drugs approved by the FDA (Food and Drug Administration) in 2021, had either one or all the following features: the genes encoding their primary assigned targets had previously been associated with the drug’s indication; proteins known to physically interact with the drug targets had established associations with the indication; some phenotypes were closely related to the drug indication and were genetically related to the drug target.

We have witnessed the development and publication of a large number of GNN-based algorithms for the prediction of drug repurposing candidates (Zeng et al. 2019, Morselli Gysi et al. 2021, Doshi and Chepuri 2022, Wang et al. 2023, Yue and Dutta 2025). The performance of these algorithms is difficult to compare due to the absence of a gold standard dataset. First, previous models [e.g. LAGCN (Yu et al. 2021), HINGRL (Zhao et al. 2022)] vary in knowledge graphs (KG), features, and training processes, making it impossible to assess whether performance differences stem from differences in GNN architectures or from data engineering choices. Second, the reported performance is often overestimated due to the absence of external validation sets, bias, and data leakage between training and validation datasets (Yang et al. 2022). Li et al. developed a benchmarking framework [HN-DREP (Li et al. 2024)] to compare methods across diverse network architectures and datasets, but because architectures were evaluated under varying hyperparameter configurations and across heterogeneous datasets, the contribution of the message-passing mechanism itself could not be isolated. Our KG-Bench framework benchmarks GNN architectural differences and hyperparameters on a fixed set of time-stamped and curated KGs. This enables fair and realistic evaluation of the performance of different message-passing mechanisms. The use of time-stamped KGs constructed from the Open Targets dataset allows models to be trained on historical data and validated or tested on future updates, simulating real-world scenarios where predictions are evaluated against newly reported drug-disease associations.

## 2 Materials and methods

### 2.1 Framework overview

**Figure 1:**
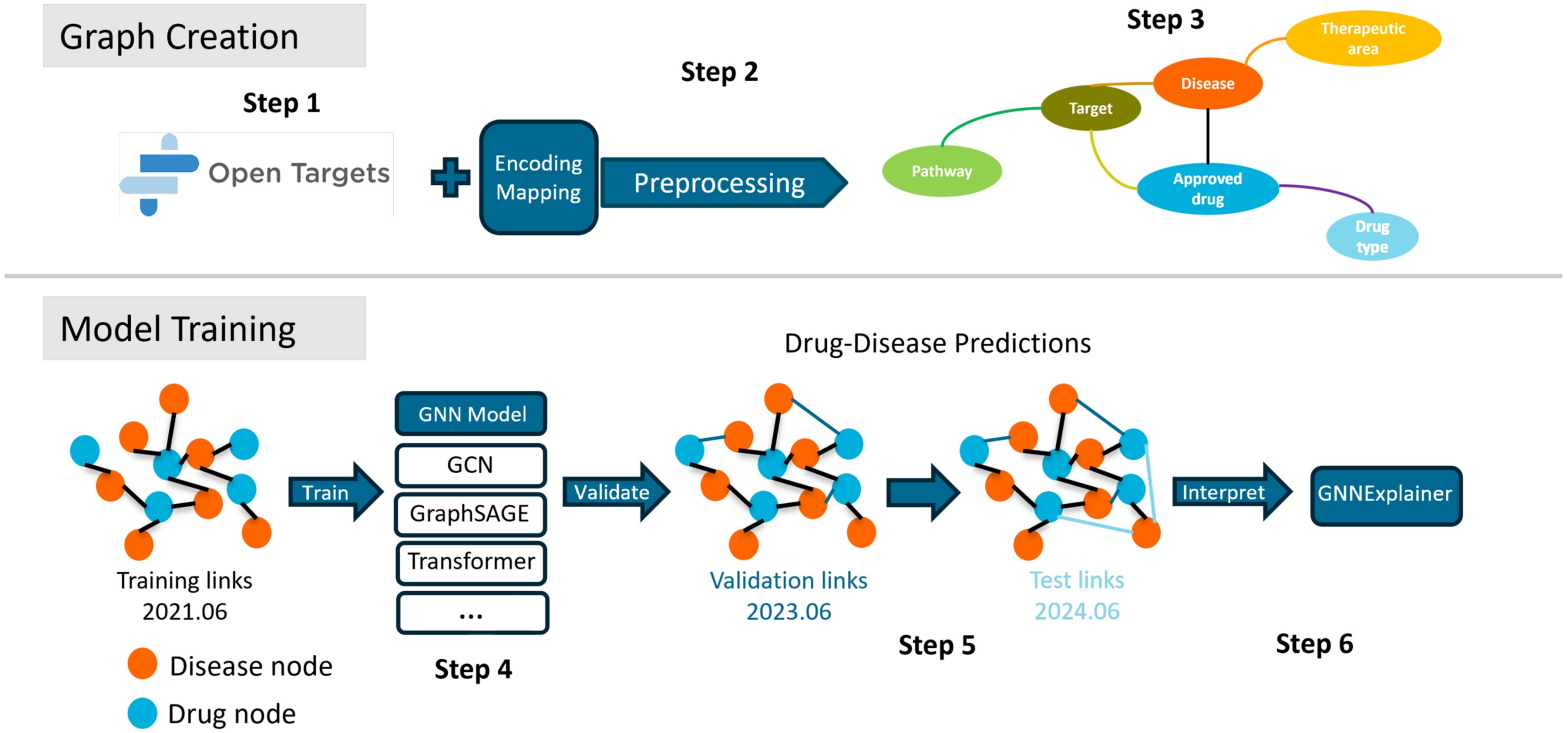
Benchmarking framework for evaluating GNN algorithms on drug repurposing using Open Targets KGs.

A graphical representation of our benchmarking framework, named KG-Bench, is provided in Fig. 1.

Step 1: The Open Targets dataset is queried to obtain the main entities and related annotations required to build the KG, followed by determining the nodes, features, and edges to extract from the graph, as described in Data Selection.

Step 2: The nodes, edges, and features are uniformly preprocessed and encoded. This involves cleaning the raw data, handling duplicates and missing values (see Section 2.2.2), normalizing column values based on their characteristics, and converting the results into tensor formats suitable for model input.

Step 3: The KG is constructed; the drug-disease edges in this version are the training set.

Step 4: We keep the nodes in the training set unchanged and focus on the drug-disease relationship edges added to these nodes in subsequent versions. We compare the number of newly added edges across OpenTargets versions and select versions with substantial increments. The newly added drug-disease edges from these versions are partitioned into validation and test sets, which are combined with the original training set to form the complete training-validation-test triplet.

Step 5: We use the KG from Step 3 to train multiple GNN models to effectively predict potential drug-disease links, and evaluate the prediction performance of the models on validation and test sets to test their generalization ability to new drug-disease relationships.

Step 6: We apply GNNExplainer to interpret the predictions by identifying key nodes and edges that influence drug-disease links. The results are statistically analyzed to assess the importance of different types of nodes and ensure the reliability of the explanation.

### 2.2 Knowledge graph

#### 2.2.1 Data selection

We first examined the parquet datasets (https://platform.opentargets.org/downloads) to identify relevant data for graph construction. Column names and data types were exported to CSV files and annotated as nodes, features, or edges based on requirements (see Section 2.2.2). We then established mappings between these entities and their attributes. The selection criteria are described below.

We established three types of core nodes: Disease, Drug and Target. In terms of drug representation, the platform adopts the molecular structure definitions used by ChEMBL, where a “parent molecule” refers to the original, unmodified form of the active ingredient, and a “child molecule” includes chemically modified variants such as salts or esters. To ensure comprehensive initial coverage of the drug space, both parent and child molecules were included. We selected only approved drug nodes, which are defined as compounds with a clinical trial max phase value of 4 for at least one indication. We further selected diseases and phenotypes that have at least one therapeutic relationship with approved drugs. In addition, we introduced three additional nodes, Drug Type, Therapeutic Area, and Pathway, to prevent some isolated triplets (Drug: Disease: Target) and increase connectivity in the KG. Among them, Drug Type is used to distinguish drug categories (e.g. small molecules, antibodies, and oligonucleotides), Therapeutic Area is used to mark disease types (e.g. infectious diseases, endocrine system diseases), and Pathway indicates the biological pathway in which the gene is located based on the Reactome platform.

The following undirected edge relationships were selected: drug-disease edges, which reflect treatment-related interactions; drug–target edges, representing the molecular targets of drugs; disease–target edges, which indicate known or predicted associations between diseases and biological targets like proteins; disease–therapeutic area edges, linking diseases to broader clinical areas; target–pathway edges, capturing the involvement of targets in biological pathways; and drug–drug type edges, specifying the classification of drugs (e.g. small molecule, antibody). The datasets and the columns selected for graph creation are listed in Table 1.

**Table 1:** Graph entities selected for input graph creation, split into node, and edge types.^a^

**Node entities**	
Dataset	Node type
Known drug	Drug
	Drug type
Target	Gene
	Pathway
Disease/phenotype	Disease
	Therapeutic area
**Edge entities**	
Dataset	Edge Type
Known drug	Drug—drug type
	Drug—target
Target	Target—pathway
Disease/phenotype	Disease—therapeutic area
Indication	Drug—disease
Association score	Disease—target

a Node types include drugs (identified by ChEMBL compound IDs), drug types (identified by drug modality categories), genes or targets (identified by Ensembl gene IDs), pathways (identified by Reactome pathway IDs), diseases (mostly identified by EFO IDs, with additional cross-references to Orphanet, MONDO, and DOID IDs), and therapeutic areas (identified by EFO IDs, indicating therapy domains).

In the graph *G (N, E)*, *N* is the number of nodes and *E* is the set of edges between nodes in the graph. Each node is represented by a one-hot encoded vector that represents its unique node type. To manage model complexity while preserving expressiveness, we selected a concise set of representative node features based on three criteria: (i) biological relevance: features must have established relevance for drug repurposing mechanisms; (ii) availability: features must be consistently available across all nodes of a given type; and (iii) computational tractability: features must be vectorizable for GNN input The feature tensor of each node type is aligned to match the size of the feature tensor of the node type with the largest number of features. The selected features of each node type are listed in Table 2. All existing edges between nodes are edges without a defined direction. This is achieved by including two sets of edges in *E* that represent interchangeable source node relationships. This allows our trained GNN to treat edges between nodes symmetrically, which is suitable for link prediction tasks.

**Table 2:** Feature definitions and encoding schemes for nodes.

Node	Column name	Feature encoding	Description
Drug	blackBoxWarning	Boolean	Whether the drug has an FDA black box warning
Drug	yearOfFirstApproval	Normalized	Year when the drug was first approved
Target	bioType	One-hot	Gene type

#### 2.2.2 Data preprocessing

The Open Targets Platform provides its datasets in Apache Parquet format, which is optimized for efficient data storage and retrieval. The Python API for Apache Arrow (https://arrow.apache.org/docs/python/index.html), PyArrow v16.0.1, was used to generate queries for extracting data from the Parquet datasets.

To build a non-redundant drug–disease KG and predict whether there is an association between disease and drug, we performed multi-step cleaning and standardization operations on the drug and disease data provided by Open Targets. In the Open Targets platform, entities such as drugs, diseases, and targets are encoded using ontology IDs. Some IDs represent high-level entities, while others are more specific entities. Inconsistent IDs will cause the same entities in different hierarchies to be treated as multiple different nodes. This may not only cause the model to learn incorrect patterns, but also bring in an imperfect separation between training, test and validation sets. It may also lead to an overestimation of the performance of algorithms.

To ensure complete separation between training, validation and test sets of drug–disease relationships, we used the drug parentId only and removed all derivative drugs representing the same chemical entity. For example, sildenafil and sildenafil citrate share the same active compound, so keeping both would lead to data leakage where the model encounters essentially the same drug in both training and test sets, resulting in overestimation of performance. Diseases that lacked therapeutic field annotations were removed, and diseases related to the broad EFO term EFO_0001444, which represents measurement, an information entity, were excluded. We further filtered out IDs with prefixes UBERON, ZFA, CL, GO, FBbt, and FMA, which represent anatomical, developmental, or cellular entities. To prevent overestimation of the performance of models, we retained only the most specific level of disease from the disease ontology and removed all parent IDs (broader disease classifications). For all filtering steps, see Code availability, the graph creation script. The Open Targets database allows users to access datasets from 1 July 2019, to the present. The graph creation script accommodates changes in column names in the different versions of the Open Targets database.

### 2.3 Graph neural network

The GNN models selected for benchmarking are:

- **GCNConv**: GCNConv is a semi-supervised learning algorithm for GNN based on a first-order approximation of spectral graph convolutions (Kipf and Welling 2017). This model aggregates and normalizes the features of neighbors of a node to update its representation. It ensures that the updated representation of each node reflects both its features and those of its neighbors, scaled by their relative importance in the graph.
- **GraphSAGE**: The GraphSAGE operator is inspired by a graph isomorphism testing algorithm (Hamilton et al. 2017). This model samples a subset of neighbors and aggregates their features, and the mean value is selected for the current task. This approach allows the model to generalize to unseen nodes by learning a function that generates embeddings based on sampled neighborhoods.
- **TransformerConv**: TransformerConv integrates GNNs with label propagation algorithms and introduces a masked label prediction strategy to prevent overfitting. This model uses attention mechanisms to focus on the most relevant neighboring nodes (Shi et al. 2021). Attention mechanisms in TransformerConv allow for a more nuanced aggregation of neighbor information compared with the other two algorithms.
- **GATConv**: Graph Attention Networks (GAT) employ learned attention mechanisms to compute edge-specific aggregation weights (Veličković et al. 2018). They learn which neighbors are most relevant for each node through multi-head attention, enabling the model to assign different importance weights across multiple representation subspaces. This adaptive weighting is particularly useful in heterogeneous drug-disease networks where the relevance of neighboring nodes varies substantially.
- **GINConv**: The Graph Isomorphism Network (GIN) operator applies learnable multi-layer perceptrons (MLPs) to neighbor aggregation, combined with an epsilon parameter that emphasizes the feature of the central node (Xu et al. 2018). GINConv is theoretically grounded in the Weisfeiler-Lehman graph isomorphism test, providing stronger graph discriminative power than simpler aggregation functions. This makes GINConv particularly effective at distinguishing diverse neighborhood structures in KGs.
- **RGCNConv**: Relational Graph Convolutional Networks (RGCN) extend graph convolutions to heterogeneous graphs by maintaining separate weight matrices for each edge type (Kipf and Welling 2017). This design allows the model to learn relationship-specific aggregation functions, enabling RGCNConv to explicitly model the distinct biological semantics of different edge types (e.g. drug–target interactions versus disease-gene associations). This capability is essential for benchmarking on KGs with multiple relation types.

For link prediction, we use an inner-product edge decoder. For a candidate drug-disease pair (u,v) with embeddings $h_{u},h_{v}\in R^{16}$, we compute a scalar logit:


$$z_{uv}=h_{u}^{⊤}h_{v},\;{\hat{p}}_{uv}=\sigma (z_{uv})=\frac{1}{1+\text{exp}(-z_{uv})}.$$

During training, we optimize the binary cross-entropy with logits:


$$L=-[y\;\text{log}\;\sigma (z_{uv})+(1-y)\;\text{log}(1-\sigma (z_{uv}))],$$

implemented with BCEWithLogitsLoss from PyTorch (https://docs.pytorch.org/) for numerical stability. Here, $z_{uv}\in R$ is the model’s predicted logit for the candidate pair (u,v), and y∈{0,1} is the ground-truth label, where y=1 indicates the pair corresponds to a known drug-disease association (positive edge) and y=0 otherwise (negative edge).

We performed a sensitivity analysis over the most influential hyperparameters: network depth (2/3/4 layers) and hidden dimension (16/64/256), across six GNN architectures. The dropout rate is fixed at 0.5 to effectively suppress overfitting, while retaining sufficient model capacity. In addition, all models apply ReLU activation functions to introduce nonlinearity, enabling the network to learn complex representations. Layer normalization is used to stabilize training and facilitate convergence. Early stopping patience, learning rate, data splits, loss function, and evaluation metrics were kept identical across architectures to isolate performance differences to model structure. Overall, these choices ensure a reasonable trade-off between model complexity, generalization ability, and reproducibility of results.

#### 2.3.1 Ablation studies

To evaluate whether specific connections between drugs and diseases affect the ability of the model to predict drug-disease associations, we shuffled the associations between drugs and diseases. In shuffling the edges between drugs and diseases, drugs were randomly reassigned to diseases while preserving both drug node degree (the number of diseases each drug is connected to) and disease node degree (the number of drugs each disease is connected to). The graph structure, negative training samples, validation set, and test set remained unchanged. To evaluate the contribution of node features, we removed all the node features or retained only the node type features.

#### 2.3.2 Negative sampling method

The Open Targets database contains only a set of positive drug: disease edges. To create a set of negative edges, we randomly sampled from the non-existent edges. We used a balanced proportion (ratio positive:negative = 1:1) for training and validation sets. To evaluate model robustness under realistic conditions, the test set was evaluated at different class imbalance proportions (ratio positive:negative = 1:1, 1:10, and 1:100).

#### 2.3.3 GNNExplainer

We used GNNExplainer from PyTorch Geometric 2.7.0 (Fey and Lenssen 2019), a perturbation-based explanation method, to identify the importance of the nodes for the drug–disease prediction model (Ying et al. 2019). This importance score quantifies how much each node (or node type) contributes to the prediction of the model. GNNExplainer optimizes learnable masks through gradient descent to identify minimal subgraphs that preserve original model predictions, thereby revealing mechanistically relevant graph components for each drug-disease pair. Comparative analysis of two neighborhood sizes was performed: 1-hop (immediate neighbors) and 2-hop (extended neighborhoods including pathway-mediated connections). For computational efficiency, we extracted k-hop subgraphs around target drug-disease pairs using subgraph sampling from PyTorch Geometric, with explanation generation performed on these reduced subgraphs before mapping results back to original node indices. Stratified random sampling was implemented at the confidence level to ensure unbiased representation. To quantify the variability and reliability of node type importance scores generated by GNNExplainer, we performed bootstrap confidence interval estimation. We generated 95% confidence intervals for node type importance rankings through bootstrap resampling (1000 iterations) of drug-disease pair explanations.

We applied a two-step validation framework to assess GNNExplainer performance. For baseline comparison, we assessed whether the model concentrates attribution on informative nodes rather than distributing importance uniformly. We generated random node-importance masks by sampling values independently on the same subgraph. We then used Mann-Whitney U tests to compare GNNExplainer’s average importance scores against this random baseline, verifying that explanations significantly differ from chance. For faithfulness testing, we determined whether top-attributed nodes are causally important for model predictions. We measured prediction performance drops after removing the top 20% of nodes by importance score. We compared these drops to those observed after removing randomly selected nodes using Wilcoxon signed-rank tests. The baseline comparison can verify that attributions differ significantly from random chance. Faithfulness testing can validate the predictive relevance of identified important nodes through systematic perturbation analysis.

## 3 Results

### 3.1 Retrospective data partitioning

**Figure 2:**
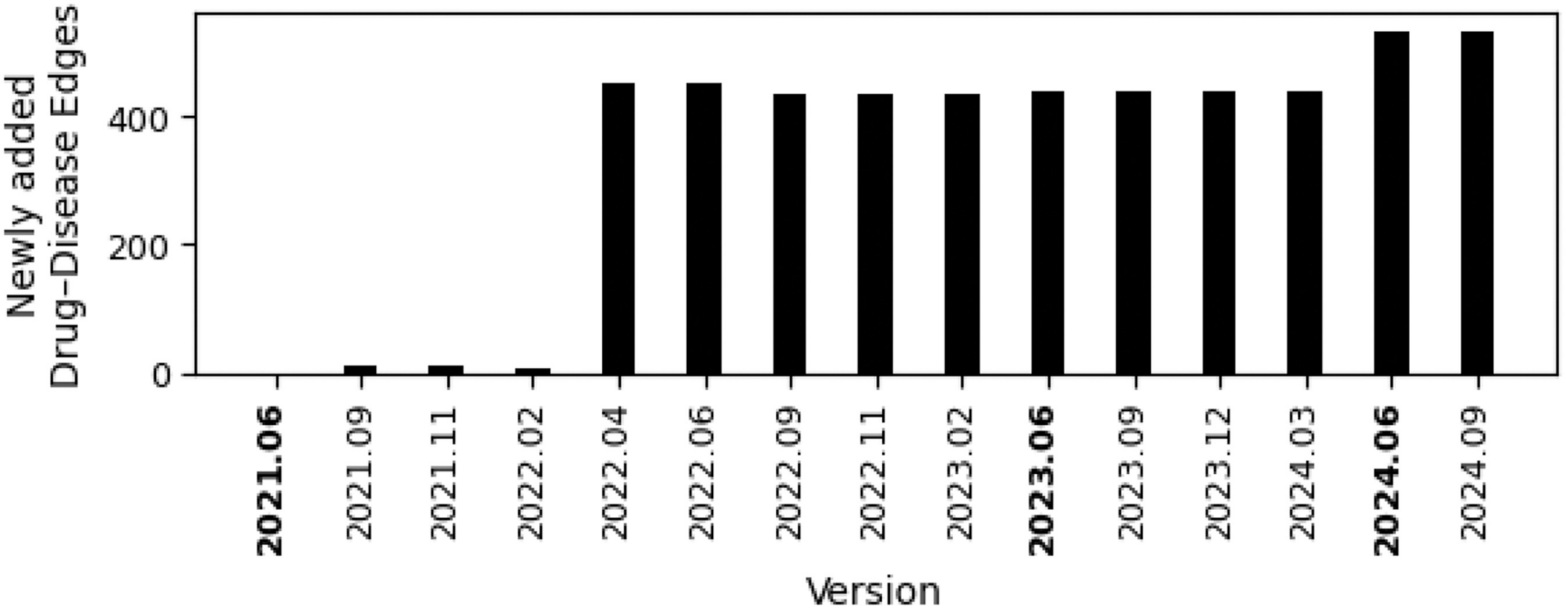
Newly added approved drug-disease associations since June 2021 across different versions of the Open Targets database.

As a training set, we selected the 2021.06 release of Open Targets, with 996 existing drug-disease edges after the strict data preprocessing criteria. Based on Fig. 2, we selected the 2023.06 release as a validation set, with just over 400 newly added drug-disease combinations. This version is more than 2 years newer than the training set and is in the middle of the dataset timeline, serving as a suitable intermediate check to assess the generalizability with increasing data availability. As a test set, we used version 2024.06. This version helps assess the prediction or generalization ability of the model in the latest real-world data, reflecting the usefulness of the GNN prediction models in future real-world scenarios.

### 3.2 Graph statistics

The Open Targets KG used for training comprises 13,004 nodes and 106,793 edges (Table 3). The graph exhibits a scale-free topology, characterized by a small number of highly connected hub nodes and a large number of sparsely connected nodes. This is reflected in the average degree of 16.42 with a high standard deviation (76.93), indicating substantial heterogeneity in node connectivity (Fig. 1, available as supplementary data at *Bioinformatics* online). The negative degree assortativity (−0.133) suggests that hubs preferentially connect to low-degree nodes, reinforcing their role as bridges across otherwise weakly connected regions. The average clustering coefficient (0.011) is very low, consistent with the sparse modularity typical of biological networks. Hub drugs and diseases are highly connected, which will accumulate richer contextual information during the message passing process of the GNN, resulting in better learned embeddings and higher prediction confidence.

**Table 3:** Summary of node and edge types in the KG, including the number of instances and feature dimension.

**Node types**		
Node type	Count	Feature dim.
Drug	1926	3
Drug type	9	1
Target	726	2
Reactome pathway	983	1
Disease	9336	1
Therapeutic area	24	1

Edge types	
Edge type	Count
Drug—drug type	1926
Drug—disease	996
Drug—target	3192
Gene—reactome pathway	3901
Disease—therapeutic area	23 801
Disease—target	72 977

### 3.3 Benchmarking GNN algorithms

**Figure 3:**
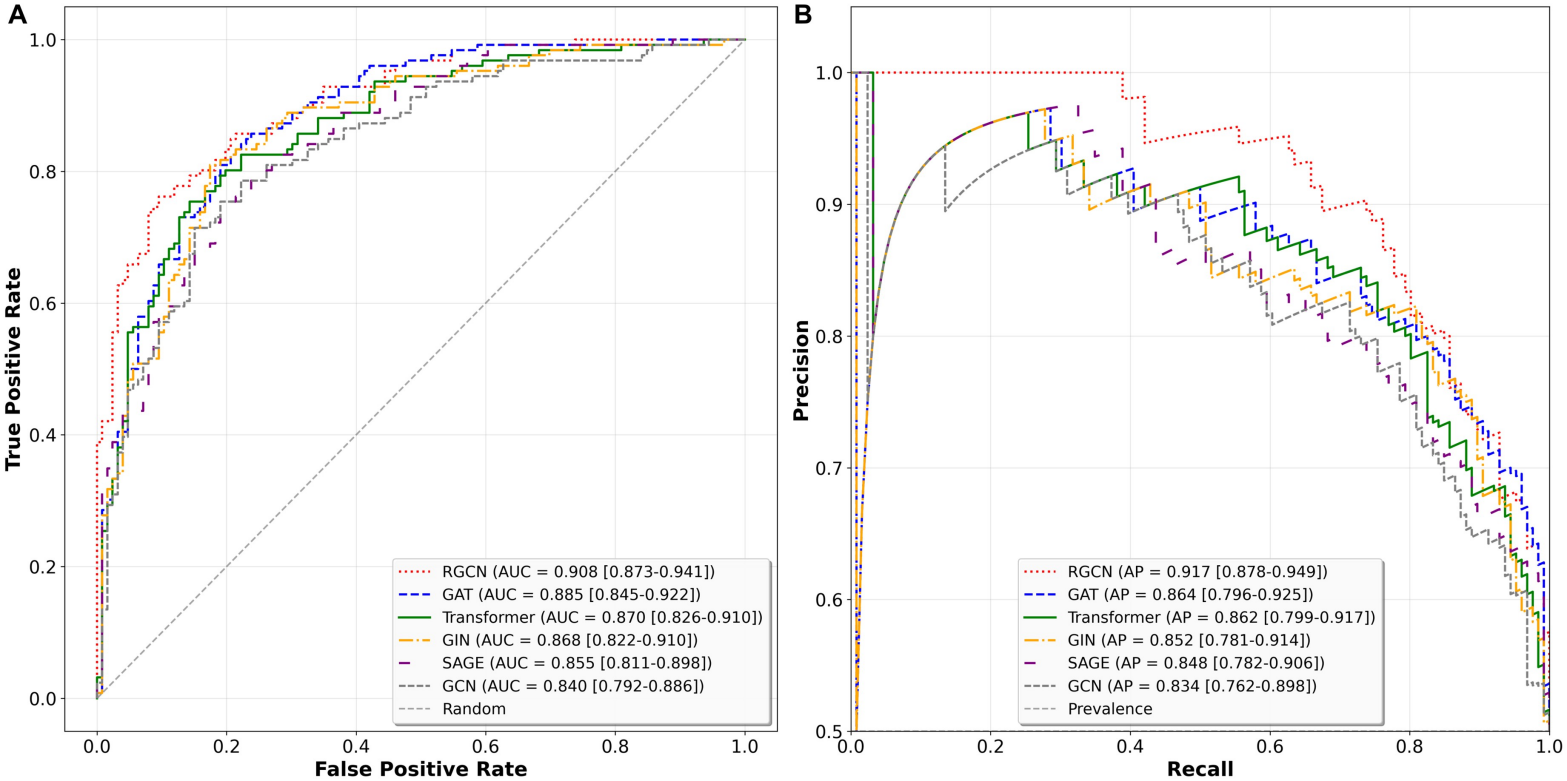
Model performance on the balanced test set evaluated with (A) ROC and (B) precision-recall curves with 95% confidence intervals. RGCN, red; GAT, blue; Transformer, green; GIN, orange; SAGE, purple; GCN, gray.

We used our benchmark KG to compare the performance of six GNN models that are commonly used in drug repurposing candidate predictions: GCNConv, TransformerConv, SAGEConv, GINConv, GATConv, and RGCNConv. To simulate real-world scenarios in which the number of drugs that do not treat a disease greatly exceeds the number of drugs indicated for that disease, we validated and tested across multiple negative proportions. The negatives were derived from a random selection of edges that were not present in the training graph. It should be noted that a small fraction of these negatives may represent effective drug-disease combinations that have not been discovered yet. Along these lines, the false positives (FPs) are the most interesting fraction for follow-up research as they may represent new drug repurposing candidates. Figure 3 shows the Receiver Operator Curves (ROC) and Precision-Recall (PR) curves of these models, with 95% confidence intervals estimated by percentile bootstrapping with 1000 resamples.

We initially explored the influence of various hyperparameter combinations on model performance for predicting drug-disease links. As shown in the sensitivity analysis (Table 1, available as supplementary data at *Bioinformatics* online), performance was consistently maximized or near-maximized with a unified configuration of three convolutional layers and 16 hidden channels across models. To ensure a fair and controlled comparison, all GNN architectures were therefore trained using this unified hyperparameter setting in the subsequent benchmarking experiments. The detailed architectural and training configurations are summarized in Table 4.

**Table 4:** Hyperparameters and architectural details for six GNN models evaluated in benchmarking experiments.

Aspect	GCN	GraphSAGE	Transformer	GAT	GIN	RGCN
Convolution type	GCNConv	SAGEConv	TransformerConv	GATConv	GINConv	RGCNConv
Num. layers	3					
Hidden channels	16					
Normalization	LayerNorm after each layer					
Activation	ReLU between layers					
Dropout rate	0.5					
Attention heads	–	–	4	4	–	–
Attention concat	–	–	No	No	–	–
MLP in conv	No	No	No	No	Yes	No
Edge type support	No	No	No	No	No	Yes
Edge decoder	Inner product ($h_{u}^{⊤}h_{v}$) → logit					
Loss function	BCEWithLogitsLoss					
Optimizer	Adam (learning rate 0.001)					

**Figure 4:**
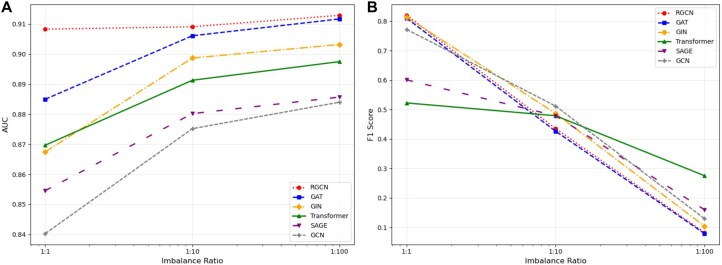
Model performance across class imbalance ratios evaluated with (A) AUC and (B) F1 score. RGCN: red, GAT: blue, Transformer: green, GIN: orange, SAGE: purple, and GCN: gray. Transformer (b) is shown with a solid line to highlight stability in real-world scenarios.

Overall, RGCN achieved the highest Area Under the Curve (AUC, 0.908) and Average Precision-Recall (APR, 0.917) across evaluation metrics, outperforming other models (Table 2, available as supplementary data at *Bioinformatics* online, Fig. 4), followed by attention-based architectures (GAT/Transformer) and MLP-based aggregation (GIN). The uniform convolution (GCN, SAGE) exhibits weaker performance. As expected, the F1 score decreases substantially as the class imbalance increases due to the challenge of maintaining precision in skewed data distributions. The TransformerConv model demonstrates remarkable stability in F1 Score across imbalance levels and achieves the highest APR in the scenario with a 1:100 ratio of true positive to true negative edges, suggesting robustness in real-world scenarios where class imbalance is common.

When interpreting the performance measures from drug repurposing prediction algorithms, FPs should not be considered as pure errors, but rather represent potential novel drug–disease associations. Analysis of FP predictions revealed significant consensus among the drug repurposing candidates predicted with the different model architectures (Fig. 2, available as supplementary data at *Bioinformatics* online).

### 3.4 Ablation studies

We performed a set of ablation studies to evaluate the contribution of features, graph structure, and node-type information on the model (Table 5). The original model makes full use of the structure and multi-node characteristics. The full input setting (all features + true links) generally provides the best performance across models, representing the effective upper bound for this task. When the edges between disease and drugs are shuffled, the performance of all models drops to a level not better than random, indicating that graph structure is crucial for prediction and that prediction based on node features alone is not enough. GCN can still make accurate predictions using graph structure only, without the inclusion of node features. Similarly, RGCN remains robust and benefits most from relational edge information. Transformer, GraphSAGE, GAT and GIN rely more on node features than GCN and RGCN. With the node-type features, the performance improves compared with the non-feature setting, indicating that mostly the node-type features, but not other node features, contribute to the prediction.

**Table 5:** Ablation study showing the impact of features and edge topology on drug–disease prediction across various GNN models.^a^

Setting	GCN		GraphSAGE		Transformer		GAT		GIN		RGCN	
	AUC	APR	AUC	APR	AUC	APR	AUC	APR	AUC	APR	AUC	APR
All feat. + true links	0.84	0.83	0.85	0.85	0.87	0.86	0.89	0.86	0.87	0.85	0.91	0.92
All feat. + shuffled links	0.50	0.51	0.47	0.49	0.45	0.48	0.48	0.50	0.48	0.49	0.51	0.52
No feat. + true links	0.83	0.79	0.49	0.48	0.42	0.46	0.25	0.40	0.61	0.62	0.86	0.87
Node type feat. + true links	0.79	0.81	0.60	0.58	0.65	0.67	0.69	0.67	0.60	0.61	0.89	0.90

a All models trained with 3 convolutional layers and 16 hidden channels. Node type feature: one-hot encoding of node type.

In the remainder of the paper, we focus on TransformerConv, which balances competitive performance with robust stability across class imbalance levels, making it better suited for our subsequent interpretability analysis.

### 3.5 Potential biases

We examined potential biases introduced by the presence of drugs in the training set and by differences in connectivity patterns. We compared prediction scores between drugs for which indications were represented in the training set versus drugs with no disease associations represented during training. When comparing prediction scores, we found that 39% of all evaluated drug–disease pairs involved drugs present in the training set, with the remaining 61% involving drugs not seen during training. The prediction score distributions were nearly identical (medians: 0.399 versus 0.394), indicating the model did not systematically bias toward or against drugs based on training set presence (Fig. 3, available as supplementary data at *Bioinformatics* online). This pattern persisted even when considering only high-confidence predictions (score $>$ 0.5), with similar median scores for training (0.647) and novel (0.649) drugs. While the model did show a tendency toward recommending well-connected drugs from the training set as candidates across a large number of diseases (up to more than 2000 disease predictions per drug), as shown in Fig. 4, available as supplementary data at *Bioinformatics* online, this connectivity preference did not translate to systematic discrimination against novel drugs. Together, these findings suggest that the model captures underlying biological connectivity patterns rather than overfitting to drugs present in the training set.

Beyond these qualitative observations, we obtained quantitative evidence from correlation analyses by examining the relationship between training frequencies and prediction scores. Drug–disease pairs showed only a weak correlation with training frequencies (Pearson *r *= 0.352, 95% CI: [0.347, 0.356], *P* <.001) (Fig. 5A, available as supplementary data at Bioinformatics online), indicating that prediction scores are not simply driven by frequency. However, log−log analysis revealed stronger rank correlation (Spearman\rho=0.569, 95%CI: [0.565, 0.572], P<.001; log−log  Pearson r=0.576,95%CI:[0.573,0.580],P<$ .001) (Fig. 5B, available as supplementary data at *Bioinformatics* online), indicating a non-linear power-law relationship. Figure 5C, available as supplementary data at *Bioinformatics* online shows that most drug-disease pairs have low combined frequencies, with a long tail of well-connected entities. These results indicate the model exhibits moderate frequency dependence for training drugs, but the weak linear correlation suggests predictions are driven by learned patterns beyond simple frequency memorization.

### 3.6 Attribution analysis

To evaluate the biological relevance of our predictions and gain insight into the reasoning of the model, we applied GNNExplainer to the TransformerConv results (Table 6). GNNExplainer analysis with different neighborhood sizes revealed significant differences in node type attribution patterns. 1-hop neighborhoods encompassed nodes with direct edges to either the target drug or disease, including immediate drug targets, drug type, disease-associated genes, and therapeutic areas. The 2-hop analysis revealed indirect relationships by including intermediate biological entities, such as shared pathways and common targets that connected drugs and diseases through one additional step. For example:


$$\text{Drug}\;\overset{1-\text{hop}}{\to }\;\text{Target}\;\text{Gene}\;\overset{2-\text{hop}}{\to }\;\text{Biological}\;\text{Pathway}$$

$$\overset{2-\text{hop}}{\leftarrow }\;\text{Disease-associated}\;\text{Gene}\;\overset{1-\text{hop}}{\leftarrow }\;\text{Disease}$$

**Table 6:** GNNExplainer-based interpretation of TransformerConv predictions across neighborhood sizes.

Metric	1-hop importance	2-hop importance
Node score range	[0.281–0.687]	[0.280–0.684]
Node type (95% CI)		
Drug	0.388 [0.385–0.391]	0.384 [0.384–0.384]
Disease	0.388 [0.383–0.393]	0.379 [0.379–0.380]
Gene	0.387 [0.387–0.388]	0.387 [0.386–0.387]
DrugType	0.586 [0.579–0.592]	0.578 [0.569–0.585]
Pathway	–	0.382 [0.382–0.383]
TherapeuticArea	0.621 [0.618–0.624]	0.617 [0.615–0.619]
Baseline comparison		
*P*-value	<.001	<.001
Faithfulness testing		
Performance drop (attributed)	0.155 ± 0.048	0.136 ± 0.052
Performance drop (random)	0.048 ± 0.018	0.051 ± 0.017
Wilcoxon *P*-value	<.001	<.001
Robustness metrics		
Hub bias correlation	0.044	0.044

The node type importance hierarchy revealed that therapeutic areas and drug types dominate predictions, reflecting the reliance of the model on higher-order drug and disease categories. Gene nodes demonstrated consistent importance across both 1-hop and 2-hop neighborhood definitions, indicating that genes maintained predictive relevance through direct associations with drugs and diseases. Pathway nodes, absent in 1-hop attributions, emerged with moderate importance (0.382) in the 2-hop analysis, providing access to network context without substantially altering gene-based predictions. This stability across neighborhood scales suggested the model captures sufficient signal through proximal relationships.

In our validation framework, baseline comparison revealed that GNNExplainer produces systematically different attribution patterns compared to random baselines (Mann-Whitney U test, P<.001). Faithfulness testing demonstrated that removing top-attributed nodes caused significantly greater performance drops than removing random nodes (2-hop: 0.136±0.052 versus 0.051±0.017; 1-hop: 0.155±0.048 versus 0.048±0.018; Wilcoxon signed-ranktest, P<.001) These results indicate that GNNExplainer produces selective attributions where high-importance nodes are causally relevant to predictions, demonstrating meaningful explanation quality with consistency across multiple neighborhood scales.

To illustrate the prediction capability of the model, we present the predicted treatment of anakinra for chronic bronchitis (confidence score: 0.911) as a case study. This prediction was chosen as it appeared among the highest-ranked drug-repurposing candidates. We systematically enumerated connection paths up to 4 steps in length using GNNExplainer, analyzing 100 paths that revealed the intermediates underlying this prediction. The most frequent intermediate node was IL1R1 (Interleukin-1 receptor type I), appearing in 100% (100/100) of identified paths. This is mechanistically coherent given anakinra’s mechanism of action as an IL-1 receptor antagonist (Baskar et al. 2016). The prominence of IL1R1 in all connection paths validates the model’s recognition of anakinra’s direct molecular target as fundamental to its therapeutic potential. Downstream of IL1R1, the model identified key pro-inflammatory mediators: IL1B (12% of the paths, the primary anakinra target cytokine) and IL6 (12% of the paths, a major acute-phase inflammatory mediator). This signature is mechanistically relevant to chronic bronchitis pathophysiology, where persistent airway inflammation driven by IL-1 and IL-6 orchestrates neutrophil infiltration, mucus hypersecretion, and airway remodeling (Rogliani et al. 2015). The pathway ANAKINRA → IL1R1 → IL1B/IL6 → chronic bronchitis has direct translational relevance. In addition, the presence of VEGFA (11% of the paths) reflects the angiogenic and airway remodeling processes that characterize the progression of chronic bronchitis. The intermediate diseases in the attribution GNNExplainer network include irritable bowel syndrome (15% of the paths), Behcet’s syndrome (10% of the paths), Hashimoto’s thyroiditis (6% of the paths), and autoinflammatory syndromes, which are all known IL-1-driven conditions in which anakinra demonstrates clinical efficacy (Dinarello et al. 2012). This coherent disease network strongly supports the inference of the model that IL-1 antagonism represents a shared therapeutic mechanism across inflammatory airway and systemic conditions. All these predictions represent a high-confidence drug repurposing candidate warranting experimental validation.

A patent (ES2766770T3, https://patents.google.com/patent/ES2766770T3/) describes the proposed use of anakinra for bronchiolitis obliterans syndrome, an inflammatory lower airway disease related to chronic bronchitis. Although this does not indicate clinical use, it provides independent mechanistic support for the model’s prediction. And importantly, such patent literature was not part of the training data.

## 4 Discussion

We presented a unified evaluation framework for GNN-based drug repurposing, dubbed KG-Bench. KG-Bench enables fair, reproducible comparison of graph-based deep learning models for the prediction of drug repurposing candidates. Our approach ensures that all benchmark components adhere to the FAIR principles: datasets are assigned persistent identifiers and registered in public repositories for findability; open-source code and comprehensive documentation are accessible from our GitHub repository; interoperability is achieved through implementation of established biomedical ontologies and unified data schemas. To ensure reproducibility and consistency across experiments, we recommend that users leverage the cleaned and preprocessed dataset provided with our framework, which includes standardized training, validation, and test splits. This setup enables direct comparison with our reported results and facilitates fair benchmarking of new models. Alternatively, researchers may choose to follow our documented preprocessing pipeline to generate their own splits, allowing for flexibility in adapting the framework to novel datasets or experimental conditions. In both cases, the modular design of our framework supports integration of custom GNN architectures and evaluation metrics, making it suitable for a wide range of drug repurposing applications.

One of the most critical contributions of this work is addressing the pervasive data leakage problem in drug repurposing benchmarks. Data leakage often occurs when related entities, such as derivative drugs or disease terms that are hierarchically related, appear across training, validation, and test sets. This leads to overly optimistic performance estimates. By removing redundant drug variants, collapsing disease identifiers with a hierarchical relationship, and ensuring strict separation of edges between training, validation and test sets, we provide a benchmark that reflects realistic prediction scenarios.

The results in Fig. 3 show performance differences among the six GNN architectures, reflecting their design principles such as attention mechanisms and neighborhood sampling strategies. RGCN achieved the highest AUC across all imbalance ratios, demonstrating superior discriminative power in heterogeneous KGs, while TransformerConv exhibited exceptional robustness in F1 scores, substantially outperforming all other architectures at high imbalance. This reveals a critical performance-reliability tradeoff: RGCN optimizes discrimination through relational inductive bias, while the attention mechanism of TransformerConv provides superior prediction calibration under class imbalance. GCN and SAGE showed the poorest performance, suggesting uniform neighborhood aggregation is insufficient for handling heterogeneous, scale-free graphs. These findings indicate that architecture selection should consider both discrimination and reliability objectives, as TransformerConv’s F1 advantage may be crucial for practical applications where prediction confidence guides clinical decision-making.

Figure 4 clearly shows the effects of different negative sampling ratios on the performance of the model. Counterintuitively, models show improved AUC performance as class imbalance increases. To understand what drives this behavior, we examine the underlying mechanisms using TransformerConv as an example (Table 3, available as supplementary data at *Bioinformatics* online). First, the positive class confidence remains stable (0.489) across all imbalance ratios, while negative class confidence slightly increases (0.809 → 0.832). This widening gap between positive and negative confidence scores creates stronger ranking separation, directly contributing to improved AUC as the imbalance increases. Second, true drug-disease associations consistently connect to hub nodes (mean degree 61.3), while randomly generated negative samples connect to progressively lower-degree nodes (17.3 → 13.4) as the imbalance ratio increases. Since high-degree nodes aggregate richer neighborhood information, positive samples leverage an amplifying structural advantage that directly explains the AUC improvement. Third, TransformerConv’s attention weighting across edge types maintains a stable ranking order of edge type prioritization (Target-Pathway > Disease−Target > Disease−TherapeuticArea > Drug − DrugType > Drug − Target > Drug-Disease) despite absolute weight fluctuations across imbalance settings. This consistent prioritization of information-rich edge types prevents signal degradation under distribution shift, enabling stable exploitation of the structural properties above. The three mechanisms demonstrate that TransformerConv’s superior ranking performance results primarily from degree-based structural advantages (mechanism 2) and confidence calibration (mechanism 1), which are stabilized by consistent attention weighting (mechanism 3).

As the ratio of negative to positive samples increased from 1:1 to 1:100, the APR dropped sharply across all models as expected (Fig. 4B). This decline highlights the sensitivity of precision to class imbalance and underscores that the choice of negative sampling strategy is a fundamental challenge in KG-based drug repurposing. Since KGs only contain known associations, it is unclear which unobserved drug-disease pairs represent true negatives and which represent pairs yet to be discovered. We chose random negative sampling in this benchmark framework for its simplicity, neutrality, and methodological consistency. Alternative strategies, such as structure-based, semantic distance-based, and ranking-based methods, have been proposed (Rendle et al. 2009, Kotnis and Nastase 2017, Tian et al. 2024), but these approaches can introduce systematic biases and may miss unexpected yet valid associations. Random sampling avoids such assumptions and ensures fair comparisons across models.

In the ablation study (Table 5), the high performance of GCN (AUC: 0.82) without node features stems from three mechanisms: (i) degree normalization $\left(1/\sqrt{d_{i}\cdot d_{j}}\right)$ provides structural bias where high-degree nodes receive different aggregation weights, (ii) this directly exploits the ground truth that positive edges connect hub nodes (mean degree 58.2 versus 14.7), enabling strong degree correlation learning (ρ = 0.687) (Fig. 6, available as supplementary data at *Bioinformatics* online), and (iii) multi-hop aggregation encodes topological position in embeddings (60.7% unique). Architectures lacking degree normalization fail correspondingly: GAT without feature diversity converges to inverted learning (ρ = −0.456, AUC = 0.245), while GraphSAGE and Transformer extract no structural signal (ρ≈ 0), degenerating to random prediction (Fig. 7, available as supplementary data at *Bioinformatics* online).

Bias is a prevalent issue in graph-based predictions of drug repurposing candidates. Models often perform better on well-studied diseases such as cancer or diabetes (Wu et al. 2022), and predictions for popular drugs with extensive literature tend to be more accurate (Perdomo-Quinteiro and Belmonte-Hernández 2024). This trend may be attributed to the higher representation frequency of these entities in the training data, which facilitates the learning of more robust embeddings and results in elevated model confidence scores. While new predictions may involve fewer connected entities or require the model to infer outside of the established hub patterns, resulting in systematically lower confidence scores. This challenge is closely related to the problem of one-shot prediction, which refers to the challenge of making accurate predictions for drugs or diseases that appear only once or very few times in the training data. Models like TxGNN (Huang et al. 2024) have addressed this kind of issue by incorporating disease similarity into the graph structure, enabling the model to generalize better to underrepresented diseases and improve prediction accuracy for novel drug-disease pairs. This bias highlights the need for bias-aware evaluation metrics. Detecting and reducing such biases is essential for ensuring fairness and generalizability in predictive models.

The types of nodes and the number of features in the benchmark KG are currently limited. Future benchmarks using the KG-Bench framework could include more features from Open Targets. Textual descriptions could be processed with language models like BioBERT (Lee et al. 2020) or Word2Vec (Church 2017) to generate text embeddings. Another promising extension to the benchmarking framework would be the implementation of edge weights, which assist the model in capturing the variable strengths of biological relationships.

To conclude, the KG-Bench framework implements a retrospective validation strategy for accurate benchmarking and provides a comprehensive performance assessment using multiple metrics, including AUC-ROC, precision–recall curves, and measures for bias and node attribution. Standardized and fair benchmarking addresses a critical gap in the field, where inconsistent methodologies and data leakage issues have complicated a fair comparison of published algorithms. Appropriate validation and benchmarking strategies will ultimately increase the proportion of drug repurposing candidates that are successful in clinical development (Ochoa et al. 2021, Wang et al. 2024).

## 5 Conclusion

Our contributions can be summarized as follows:

- **Open Targets Knowledge Graph:** We constructed a harmonized biomedical KG from Open Targets, integrating drugs, diseases, targets, and contextual annotations, and applied strict preprocessing to prevent data leakage from hierarchical ontology.
- **GNN Benchmarking:** We systematically evaluated multiple GNN architectures under consistent conditions, including interpretability analysis and ablation studies.
- **Framework Usability:** New users can either use the provided benchmark data for direct model comparison or reuse the preprocessing pipeline to generate custom datasets for their own models.

## Supplementary Material

btag159_Supplementary_Data

## Data Availability

The code and data used in this study is available at https://github.com/cmbi/Benchmark_GNN_OpenTargets
