# Supplementary material for: KG-bench: benchmarking graph neural network algorithms for drug repurposing
Source: Bioinformatics. 2026 May 8;42(5):btag159. doi: 10.1093/bioinformatics/btag159 (PMC13171177; doi:10.1093/bioinformatics/btag159)
Supplement: btag159_Supplementary_Data [file btag159_supplementary_data.zip › Appendix.pdf]

Appendix: List of investigators from the SIMPATHIC consortium

Partner 1: Amsterdam University Medical Centers (Amsterdam UMC)

Tessa Braam, Esther Bührman, Juliette Chevalier, Martina Cornel, Lotte Haverman, Vivi Heine, Annelieke Müller, Tessel Rigter, Tom Steenbergen, Clara van Karnebeek, Hans Waterham

Partner 2: Asociación de Síndrome Miasténico Congénito (ASMIC)

Ignacio Escuder Bueno

Partner 3: Biovista

Leda Persidi, Eftychia Lekka, Andreas Persidis, Vassilis Virvilis

Partner 4: Charité – Universitätsmedizin Berlin

Markus Schülke-Gerstenfeld

Partner 5: Children's Hospital of Eastern Ontario (CHEO)

Emily Freeman, Haley Geertsma, Hanns Lochmuller, Alex Mackenzie, Kaela O'Connor, Izabella Pena, Sally Spendiff, Rachel Thompson

Partner 6: EATRIS – European Advanced Translational Research Infrastructure in Medicine

Martin de Kort, Anna Sanchez, Cansu Tekin

Partner 7: Erasmus University Rotterdam

Francisca Vargas Lopes

Partner 8: EUPATI – European Patient Academy on Therapeutic Innovation

Larisa Aragon Castro, Mandy Daly, Maria Dutarte, Jana Popova, Enrico Tricanico

Partner 9: EURO-ATAXIA – European Federation of Hereditary Ataxias

Julie Greenfield, Andreas Nadke

Partner 10: EURO-DYMA – European Dystonia and Myopathy Association

Peter Ashley, Alain Geille, Christiana Vasileiadi

Partner 11: Fair Data Systems

João Cardoso, Eduardo Quemada, Mark Wilkinson

Partner 12: Familias GA

Albert Carbonell

Partner 13: Fondation Maladies Rares

Laura Benkemoun, Magda Granata, Pascale Milani, Chloe Morel, Daniel Scherman

Partner 14: Heinrich-Heine-Universität Düsseldorf

Judith Hatzfeld, Selene Lickfett, Alessandro Prigione, Isabella Tolle

Partner 15: Heidelberg University Hospital

Elena Boyd, Samuele Cesaro, Florian Gleich, Samuel Hofmann, Sabine Jung-Klawitter, Stefan Kölker, Christian Thiel

Partner 16: International Mito Patients

Jo de Bry

Partner 17: National Centre for Scientific Research Demokritos

Fotis Aisopos, Anastasia Krithara, Anastasios Nentidis, George Paliouras, Stavroula Svolou

Partner 18: Radboud University Medical Center (Radboudumc)

Merel Adjobo-Hermans, Hans van Bokhoven, Hilde Braakman, Baziel van Engelen, Alex Garanto, Elena Garcia Lara, Joanna in 't Hout, Martijn Huijnen, Mirian Janssen, Werner Koopman, Dirk Lefeber, Kris Leeuwenberg, Karlien Mul, Nael Nadif Kasri, Kit Roes, Raymond Schipper, Vedrana Stefanic, Steven Teerenstra, Peter-Bram 't Hoen, Nicol Voermans, Bart van de Warrenburg, Rick Wansink, Siqi Wei, Ka Man Wu, Jurriaan Zwier

Partner 19: SSADH Association

Claudio Cinquemani

Partner 20: United Mitochondrial Disease Foundation (UMDF)

Philip Yeske

Partner 21: University of Lisbon

Adelaide Maria Fernandes Borralho, Maria Martins, Sara Pintado

Partner 22: University of Ljubljana

Rok Dreu, Iztok Grabnar, Jakob Kolar, Zoran Lavric, Igor Locatelli, Irena Mlinaric

Partner 23: University of Luxembourg

Ibrahim Boussaad, Vyron Gorgogietas, Rejko Krueger, Alexia Tiberi

Partner 24: University of Tübingen

Holm Graessner, Charlotte Kogel, Olaf Riess, Birte Zurek

Partner 25: University Hospital Tübingen

Priscila Pereira Sena, Thorsten Schmidt

Partner 26: Volwassenen, Kinderen en Stofwisselingsziekten (VKS)

Hanka Dekker
