# Supplementary material for: KG-bench: benchmarking graph neural network algorithms for drug repurposing
Source: Bioinformatics. 2026 May 8;42(5):btag159. doi: 10.1093/bioinformatics/btag159 (PMC13171177; doi:10.1093/bioinformatics/btag159)
Supplement: btag159_Supplementary_Data [file btag159_supplementary_data.zip › Supplementary_materials.pdf]

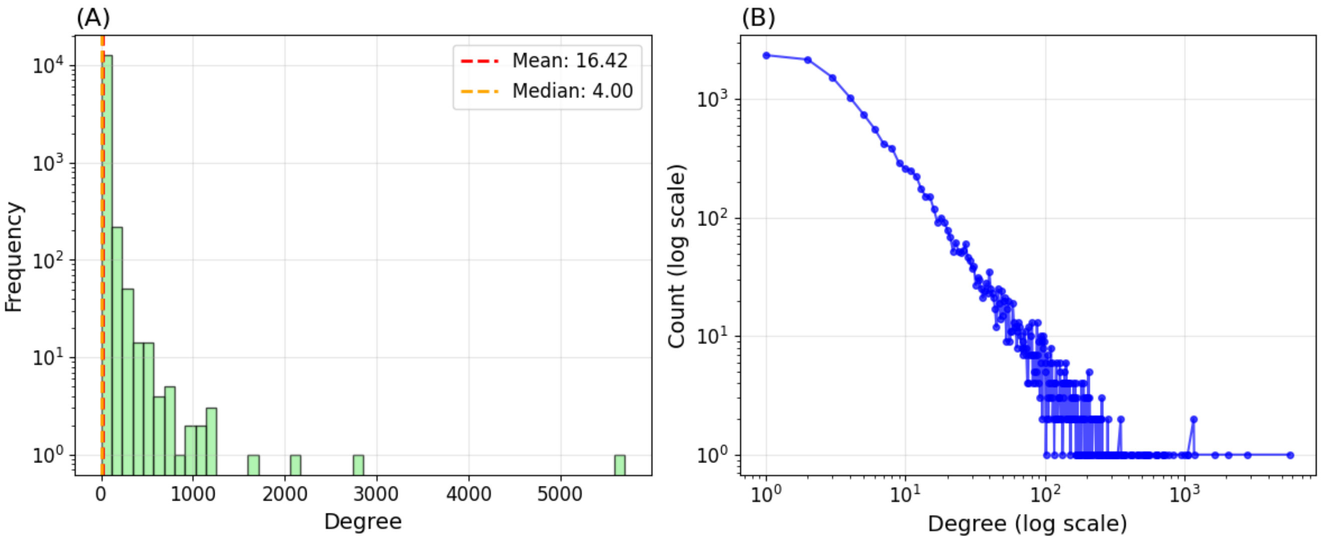

Figure S1 Graph topology of the Open Targets KG. (A) Degree frequency distribution (log scale) demonstrates large heterogeneity in node connectivity. The extreme right tail is dominated by top-level therapeutic area ontology nodes. The highest-degree node (degree = 5,698) corresponds to OTAR_0000018 ("Genetic, Familial or Congenital Disease"), a broad umbrella term in the OpenTargets EFO-derived disease ontology whose high connectivity reflects the large number of diseases classified beneath it. (B) A power-law relationship in log-log space indicates a scale-free graph structure.

Table S1. Hyperparameter sensitivity analysis across six GNN architectures. Each architecture was evaluated across nine configurations obtained by systematically varying the number of layers (2, 3, 4) and hidden dimensions (16, 64, 256). All metrics are reported on the validation set (OpenTargets v23.06).

Model	Layers	Hidden	AUC	APR	F1
GCNModel	**4**	**16**	**0.885**	**0.889**	**0.833**
	3	16	0.882	0.896	0.815
	2	256	0.865	0.886	0.802
	2	16	0.835	0.844	0.727
	3	64	0.809	0.824	0.712
	4	64	0.793	0.804	0.730
	4	256	0.793	0.799	0.743
	2	64	0.758	0.790	0.671
	3	256	0.742	0.740	0.712
SAGEModel	**3**	**16**	**0.890**	**0.883**	**0.820**
	2	16	0.881	0.870	0.812
	4	64	0.879	0.889	0.811
	2	64	0.853	0.841	0.772
	3	64	0.847	0.833	0.777
	2	256	0.833	0.815	0.779
	4	16	0.784	0.778	0.746
	4	256	0.775	0.728	0.715
	3	256	0.721	0.661	0.716
TransformerModel	**4**	**64**	**0.906**	**0.916**	**0.818**
	2	16	0.905	0.907	0.819
	3	16	0.902	0.910	0.836
	2	64	0.900	0.903	0.810
	3	64	0.892	0.899	0.811
	2	256	0.889	0.894	0.801
	4	256	0.807	0.744	0.759
	3	256	0.615	0.519	0.632
	4	16	0.598	0.610	0.564
GATModel	**2**	**16**	**0.918**	**0.918**	**0.845**
	3	16	0.911	0.916	0.845
	4	64	0.883	0.896	0.818
	2	256	0.874	0.879	0.817
	2	64	0.868	0.880	0.806
	3	64	0.846	0.846	0.797
	3	256	0.836	0.843	0.746
	4	256	0.815	0.809	0.762
	4	16	0.677	0.621	0.635
GINModel	**3**	**16**	**0.907**	**0.915**	**0.849**
	3	64	0.904	0.912	0.845
	4	256	0.901	0.908	0.839
	4	64	0.889	0.899	0.835
	2	256	0.885	0.888	0.821
	3	256	0.855	0.863	0.782
	2	64	0.832	0.836	0.769
	4	16	0.723	0.726	0.691
	2	16	0.730	0.720	0.678
RGCNModel	**3**	**16**	**0.872**	**0.891**	**0.798**
	4	16	0.857	0.880	0.781
	2	16	0.849	0.866	0.759
	2	256	0.836	0.861	0.732
	2	64	0.807	0.806	0.760
	3	64	0.817	0.816	0.768
	4	64	0.776	0.750	0.713
	3	256	0.772	0.757	0.717
	4	256	0.749	0.723	0.711

Table S2. Performance of GNN architectures across class imbalance ratios (mean and 95% confidence interval). TransformerConv shows the highest F1 at the ratio of 1:100.

Model	Ratio 1:1			Ratio 1:10			Ratio 1:100		
	AUC	APR	F1	AUC	APR	F1	AUC	APR	F1
GCNConv	0.84 [0.79–0.89]	0.83 [0.76–0.90]	0.77	0.88 [0.84–0.91]	0.56 [0.47–0.64]	0.51	0.88 [0.84–0.92]	0.20 [0.14–0.28]	0.13
SAGEConv	0.85 [0.81–0.90]	0.85 [0.78–0.91]	0.60	0.88 [0.85–0.91]	0.51 [0.42–0.60]	0.48	0.89 [0.86–0.91]	0.16 [0.10–0.23]	0.16
TransformerConv	0.87 [0.83–0.91]	0.86 [0.80–0.92]	0.52	0.89 [0.86–0.92]	0.58 [0.49–0.67]	0.48	0.90 [0.87–0.93]	0.20 [0.14–0.28]	**0.28**
GATConv	0.89 [0.85–0.92]	0.86 [0.80–0.92]	0.81	0.91 [0.88–0.93]	0.58 [0.49–0.67]	0.43	0.91 [0.89–0.93]	0.19 [0.14–0.27]	0.08
GINConv	0.87 [0.82–0.91]	0.85 [0.78–0.91]	0.81	0.90 [0.87–0.93]	0.57 [0.49–0.67]	0.49	0.90 [0.87–0.93]	0.17 [0.12–0.25]	0.10
RGCNConv	**0.91** [0.87–0.94]	**0.92** [0.88–0.95]	**0.82**	**0.91** [0.88–0.94]	**0.65** [0.56–0.73]	0.44	**0.91** [0.88–0.94]	**0.21** [0.16–0.29]	0.08

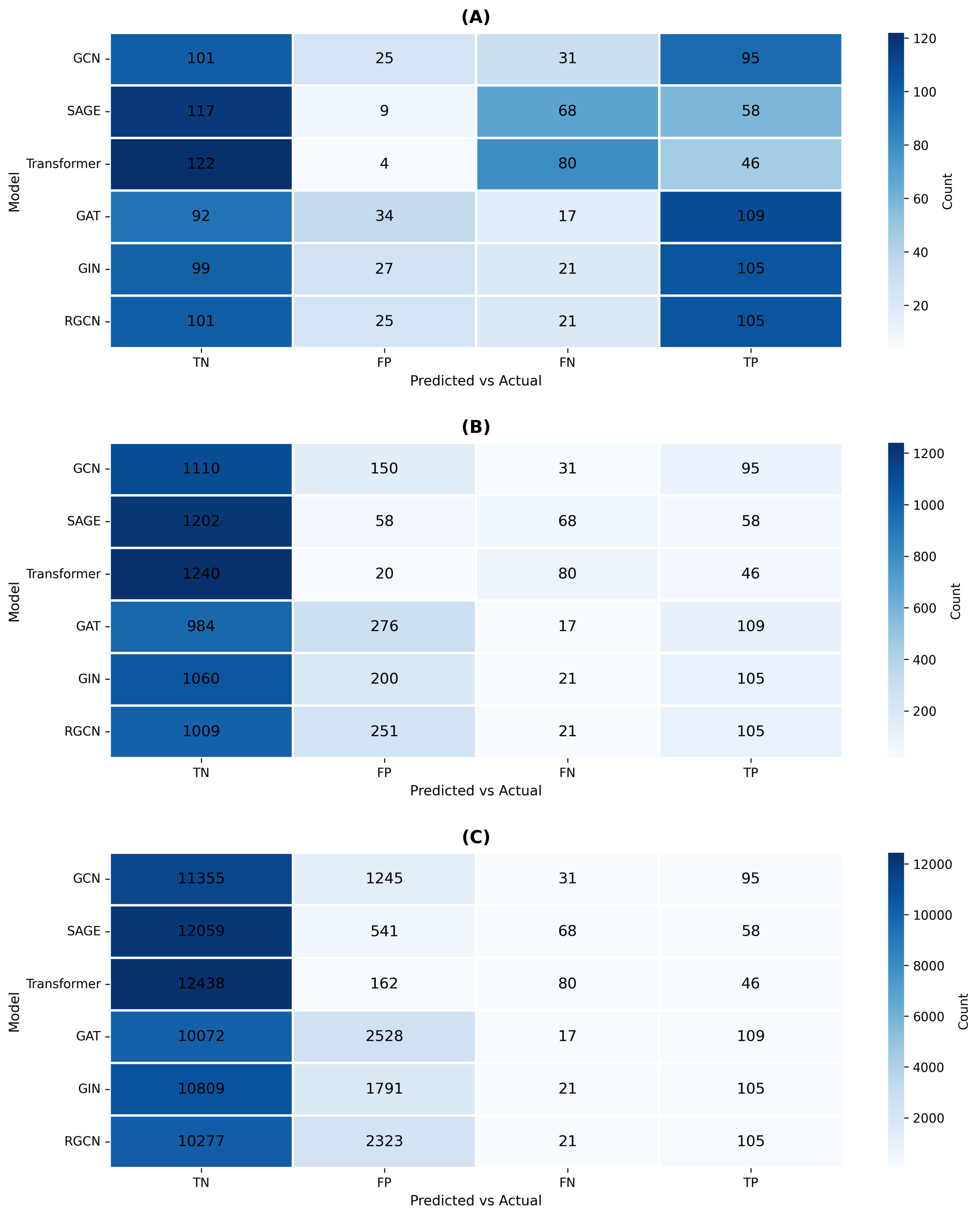

Figure S2 Confusion matrix counts (TN, FP, FN, TP) for six GNN models on the test set. Each panel corresponds to a model trained separately under a specific positive:negative ratio: (A) 1:1, (B) 1:10, and (C) 1:100.

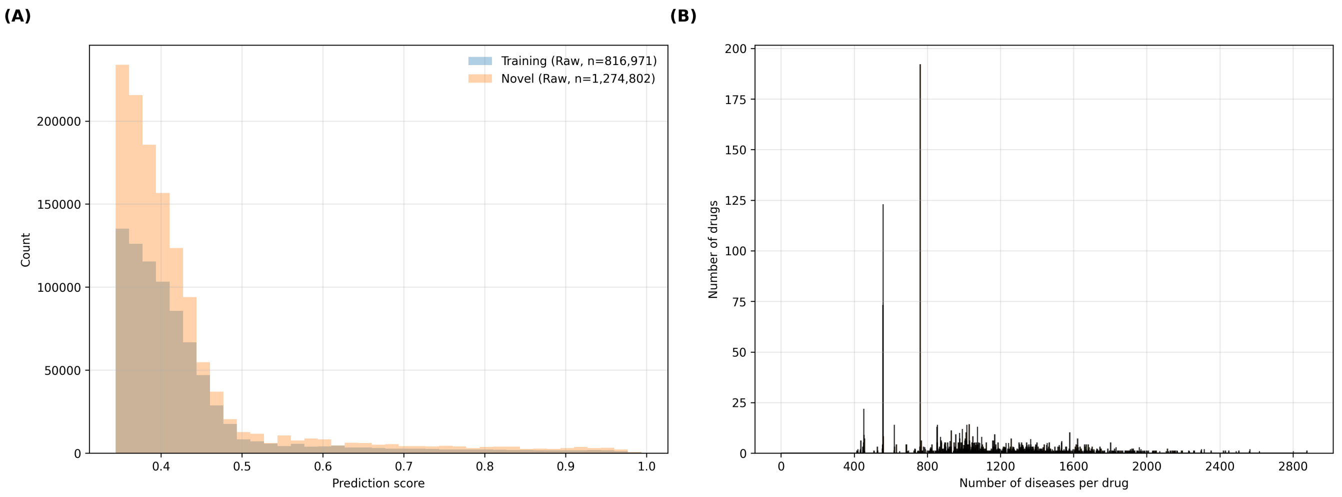

Figure S3 Bias diagnostics of model predictions from the TransformerConv model. (A) Distributions of model prediction scores, comparing links involving drugs present in the training set (blue) with those involving drugs absent from the training set (orange). (B) Frequency distribution of drug connectivity in predictions. X-axis shows the number of diseases per drug; Y-axis shows the count of drugs with that connectivity level.

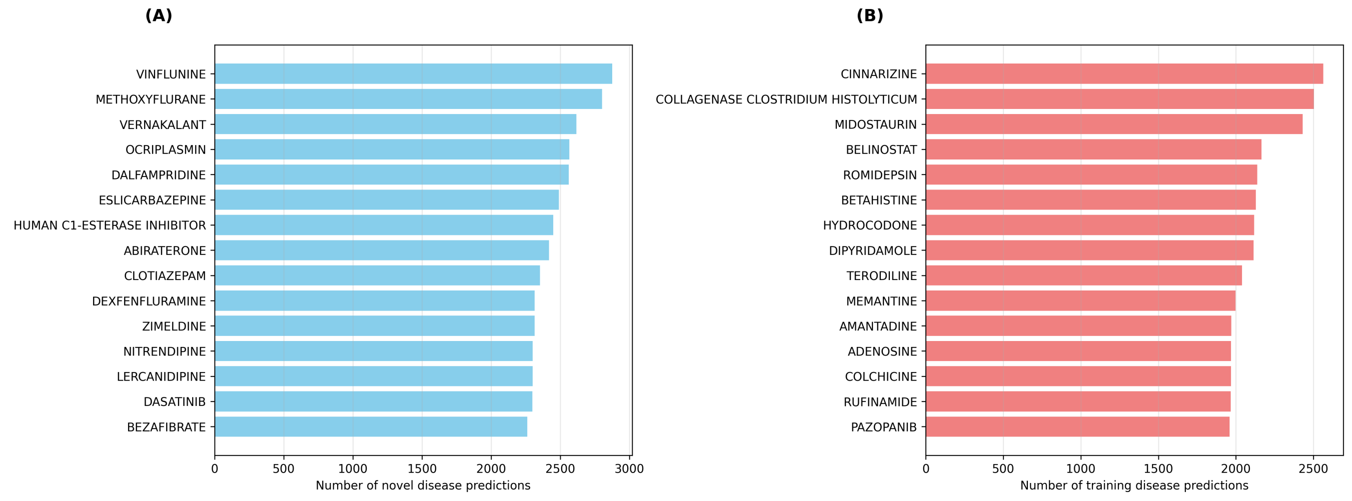

Figure S4 Analysis of model bias through drug-level prediction patterns. (A) Drugs absent from the training set were ranked by the total number of disease predictions they involve. (B) Drugs present in the training set ranked by the total number of disease predictions they involve.

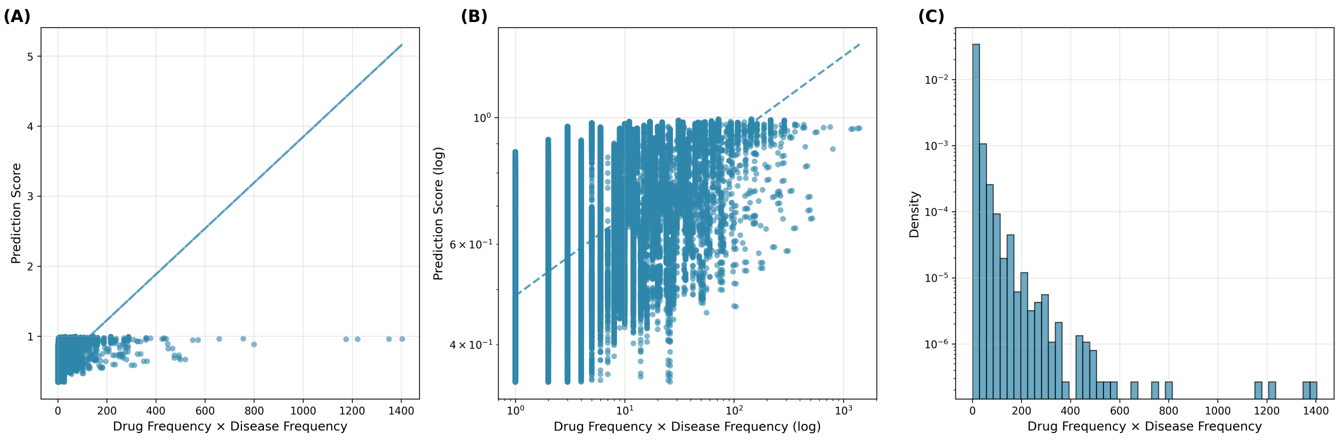

Figure S5 Correlation between model prediction scores and the combined training frequency of drug–disease pairs. (A) Scatter plot showing raw prediction scores versus the product of drug and disease frequencies in the training set. (B) Log–log scatter plot highlighting the heavy-tailed relationship between frequency and score. (C) Distribution of combined training frequencies across all evaluated pairs, illustrating the skewed connectivity pattern in the KG.

Table S3. Mechanistic analysis of the performance of the Transformer model under different class imbalance ratios

(A) Mechanism 1: TransformerConv achieves superior auc by maintaining stable positive confidence while increasing negative confidence, creating stronger ranking separation.

Imbalance Ratio	Positive Confidence	Negative Confidence	AUC
1:1	0.489	0.809	0.870
1:10	0.489	0.826	0.891
1:100	0.489	0.832	0.898

(B) Mechanism 2: True drug-disease associations exploit preferential connection to high-degree hub nodes, while random negatives connect to progressively lower-degree nodes, amplifying structural advantage as class imbalance increases.

Imbalance Ratio	Pos Mean Degree	Neg Mean Degree	AUC
1:1	61.300	17.300	0.870
1:10	61.300	13.900	0.891
1:100	61.300	13.400	0.898

(C) Mechanism 3: The attention mechanism of TransformerConv maintains consistent prioritization of information-rich edge types across all class imbalance settings, stabilizing exploitation of structural advantages.

Edge Type	1:1	1:10	1:100
Target–Pathway	0.210	0.294	0.183
Disease–Target	0.041	0.063	0.065
Disease–TherapeuticArea	0.033	0.041	0.036
Drug–DrugType	0.037	0.031	0.024
Drug–Target	0.027	0.020	0.016
Drug–Disease	0.018	0.014	0.013

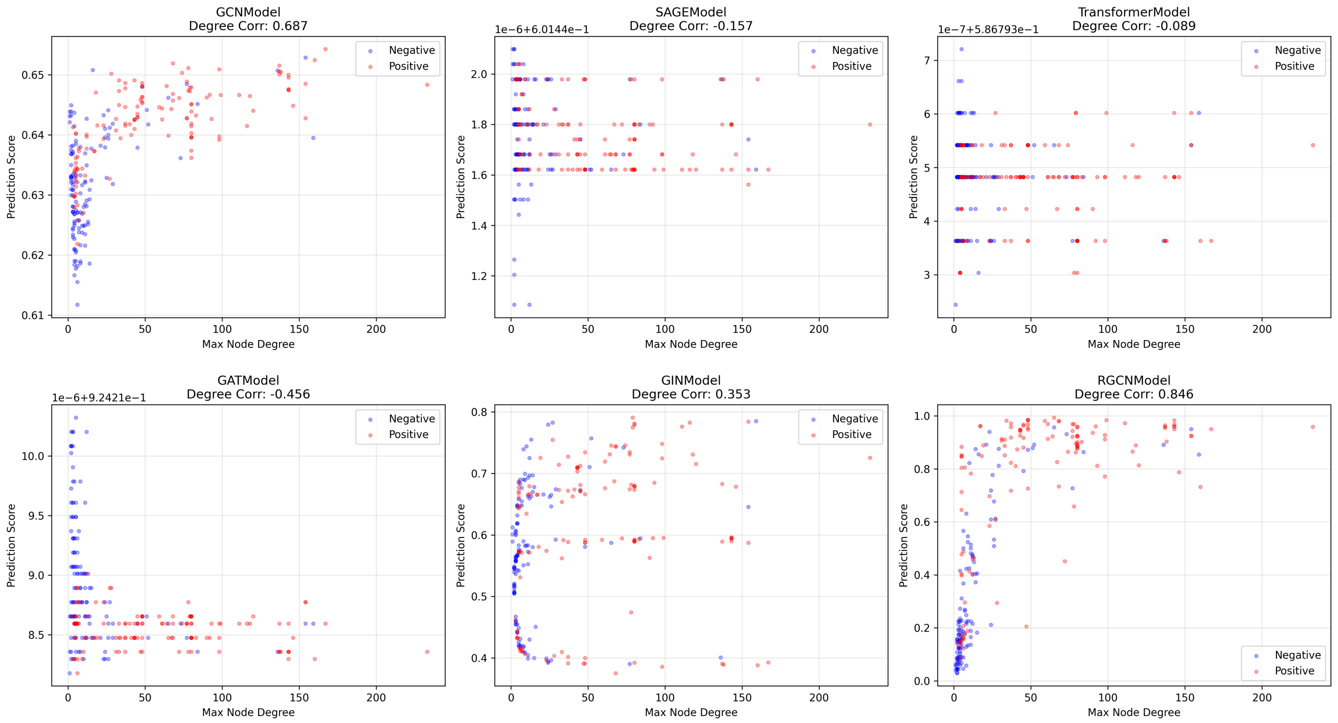

Figure S6 Degree bias reveals how GNN architectures learn from graph structure. GCN (ρ=0.687) and RGCN (ρ=0.846) show strong positive correlations: positive edges (red) concentrate at high-degree nodes (50-200) while negative edges (blue) concentrate at low-degree nodes (0-50), demonstrating successful exploitation of hub structure. GAT exhibits inverted learning (ρ=-0.456), assigning high predictions to low-degree nodes. GraphSAGE (ρ=-0.157) and Transformer (ρ=-0.089) show no degree correlation, indicating random predictions. This visualization demonstrates that degree normalization is the key architectural feature enabling learning from topology without node features.

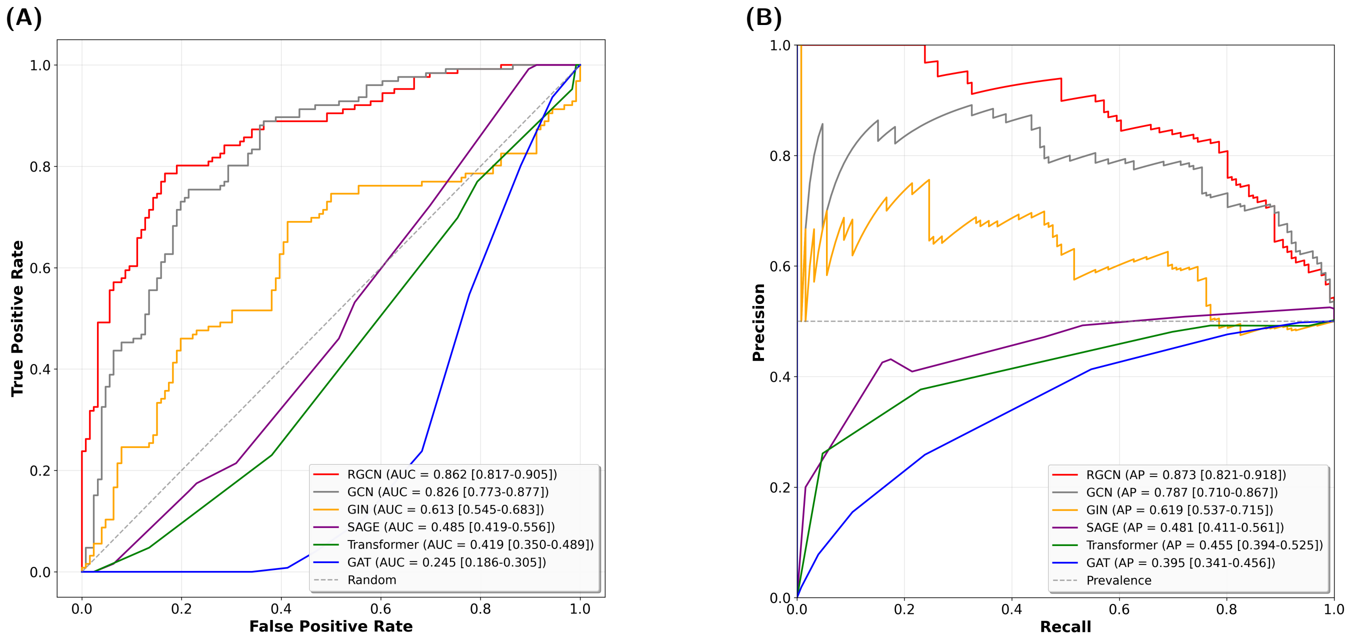

Figure S7 Model performance on test set without features, evaluated with (A) ROC and (B) Precision-Recall curves with 95% confidence intervals. RGCN: red, GAT: blue, Transformer: green, GIN: orange, SAGE: purple, and GCN: gray.
